# Cytotoxic efficacy of filanesib and melphalan combination is governed by sequence of treatment in human myeloma cells

**DOI:** 10.1038/bcj.2016.92

**Published:** 2016-10-07

**Authors:** E J Norris, D DeStephanis, B Tunquist, S Usmani, R Ganapathi, M Ganapathi

**Affiliations:** 1 Department of Cancer Pharmacology, Levine Cancer Institute, Carolinas HealthCare System, Charlotte, NC, USA; 2 Array BioPharma Inc., Boulder, CO, USA; 3 Department of Hematologic Oncology and Blood Disorders, Levine Cancer Institute, Carolinas Healthcare System, Charlotte, NC, USA

The integration of novel agents (for example, proteasome inhibitors, IMiDS) into frontline therapy for multiple myeloma (MM) has significantly improved response rates and increased progression-free survival. Nevertheless, most patients with MM relapse often with resistant disease.^1^ Several clinical trials assessing the current role of high-dose chemotherapy (HDT) with autologous stem cell transplant (ASCT) have reaffirmed the utility of HDT-ASCT for treating transplant-eligible MM patients.^2^ Efforts to improve the standard conditioning regimen (single agent melphalan 200 mg/m^2^) for ASCT have generally failed due to increased toxicity without the demonstration of superiority.^3^ However, recent evidence suggests that conditioning regimens combining HDT-melphalan with novel agents (bortezomib, lenalidomide) are safer and improve patient response and outcome.^4, 5^

Filanesib (ARRY-520) is a first-in-class, small-molecule inhibitor of Kinesin Spindle Protein (KSP) with activity in MM refractory to standard of care agents. Filanesib inhibits mitotic spindle pole separation in proliferating cells, leading to mitotic arrest and apoptosis *in vitro* and *in vivo*.^6, 7^ Clinically, filanesib was well tolerated and induced partial responses or better in heavily pretreated patients with advanced MM.^8^ Although non-hematologic toxicities were rare, filanesib was associated primarily with cytopenias.^7, 9^ *In vitro* evidence of a synergistic interaction between filanesib and proteasome inhibitors or filanesib and IMiDS led to the initiation of ongoing phase 2 clinical trials, investigating the effect of filanesib in combination with carfilzomib (Clinicaltrials.gov NCT01989325) or pomalidomide/dexamethasone (Clinicaltrials.gov NCT02384083) in patients with advanced MM. To date, a combination of filanesib and melphalan as a potential intensive conditioning regimen has not been explored. Therefore, the objective of this study was to assess the cytotoxic effects of combination melphalan and filanesib *in vitro*.

The cytotoxic effects of melphalan and filanesib were assessed by MTS assay in three human myeloma cell lines (HMCLs) with differing intrinsic sensitivities to these agents (pan-sensitive MM1S, filanesib-resistant NCIH929 and melphalan-resistant U266 cells). Continuous treatment with melphalan alone (10 μM) for 72 h inhibited cellular proliferation of U266, MM1S and NCIH929 cells by 16, 58 and 55%, respectively (Figure 1a). Treatment with filanesib alone for 72 h led to a dose-dependent inhibition, with 50% inhibition observed at ~5 nM (U266), 1.0 nM (MM1S) and 1.6 nM (NCIH929). Unexpectedly, an antagonistic interaction, as determined by the Chou-Talalay method,^10^ between filanesib and melphalan was observed in all cell lines with the exception of an additive effect observed with 1 nM filanesib in MM1S. Calculated combination index (CI) values (values <0.9 indicate synergism, values >1.1 indicate antagonism) of melphalan (10 μM) and filanesib (5 nM) were 15.45, 1.86 and 2.36 for U266, MM1S and NCIH929, respectively. Although the cytotoxic effect of combination treatment in U266 cells was greater than melphalan alone, it was significantly less cytotoxic than filanesib alone at doses greater than 1 nM. This effect was not observed in MM1S and NCIH929.

Since melphalan induces accumulation of cells in S-phase, whereas filanesib is associated with accumulation in the G2M phase of the cell cycle, the antagonistic interaction between melphalan and filanesib may be due to cell cycle traverse perturbations. Therefore, we investigated the effect of sequential treatments, involving: (1) treatment with melphalan first for 1 h followed by addition of filanesib (Mel→Fil) and (2) treatment with filanesib for 24 h followed by addition of melphalan (Fil→Mel). For both sequences, the total incubation time was 48 h. As shown in Figure 1b, filanesib induced significantly higher apoptosis than melphalan alone (70 vs 53%, *P*=0.017) and apoptosis induced by Fil→Mel treatment was significantly higher than that induced by Mel→Fil (67 vs 53%, *P*=0.034). However, the apoptotic response of neither combination exceeded that of individual agents, and in the case of Mel→Fil the apoptotic response was inferior to that of filanesib alone. Apoptosis induced by filanesib alone and Fil→Mel treatment was accompanied by decreased expression of the anti-apoptotic proteins MCL-1 and BCL-2. Downregulation of MCL-1 was similar in magnitude between filanesib and Fil→Mel, whereas BCL-2 levels were lower, albeit modestly, in Fil→Mel compared with filanesib in all three replicates (Figure 1c). Conversely, Mel→Fil treatment attenuated filanesib-induced downregulation of BCL-2 and MCL-1. Expression of these proteins was unaffected by melphalan alone. The differences between the induction of apoptosis in Mel→Fil- and Fil→Mel-treated cells correlated with the effects on cell cycle distribution. Mel→Fil-treated cells primarily accumulate in S-phase compared with an accumulation in late-S/G2M in Fil→Mel-treated cells (Figure 1d). Taken together, the data suggest that melphalan-induced cell cycle arrest in S-phase prevents progression of cells through mitosis, and attenuates downregulation of key survival proteins, which is required for filanesib-induced apoptosis.

Although the preliminary evaluation of drug sequencing was informative, in order to establish superiority of sequence specific differences with filanesib and melphalan, cell recovery following sequential treatments in drug-free media was tested. Cells were treated for 1 h with melphalan followed by 48 h of filanesib (Mel→Fil) or inversely with 48 h filanesib followed by 1 h of melphalan (Fil→Mel). At the end of treatment, cells were washed, resuspended in drug-free media, and allowed to recover for 7 days. Forty-eight hours after drug washout, a significant increase in the percentage of cells staining positive for Annexin V, an early marker of apoptosis, was observed following treatment with either melphalan or filanesib in all cell lines (Figure 2a). Induction of apoptosis was enhanced by Fil→Mel treatment, while no increase was observed with Mel→Fil treatment. Accordingly, on recovery day 3, cell proliferation was significantly lower for Fil→Mel compared with Mel→Fil (Figure 2b). This effect persisted through day 7 as no cell proliferation was observed in the Fil→Mel group from day 3 to day 7, while the Mel→Fil treatment group began to recover. Two-way ANOVA analysis of each treatment protocol revealed a synergistic interaction between melphalan and filanesib on day 7 when filanesib treatment preceded melphalan. Direct comparison of cell survival at day 7 between the two combination treatments demonstrated a 2.9- (U266), 17.6- (MM1S) and 6.5- (NCIH929) fold reduction in cell growth for the Fil→Mel treatment compared with Mel→Fil, demonstrating superiority of the Fil-Mel sequential drug schedule.

The results reported in this study suggest that the interaction between filanesib and melphalan is dependent on the sequence of treatment. Exposure to melphalan prior to filanesib is associated with cell cycle arrest in S-phase and inhibition of filanesib induced apoptosis. Indeed, similar results were observed in previous clinical studies with combination of paclitaxel and cisplatin.^11^ However, our observation demonstrating a synergistic interaction when cells are treated with filanesib prior to melphalan represents a potential novel conditioning regimen, especially in patients with refractory and/or relapsed disease.^4, 12^ Since known negative prognostic factors for early relapse after ASCT include high plasma cell proliferative index at diagnosis and p53 status (del17p),^13, 14^ Fil→Mel combination treatment could improve outcomes in this subgroup due to targeting of proliferative plasma cells and melphalan-resistant p53 mutant (del17p) cells by filanesib.^14, 15^ Indeed, U266 cells that lack functional p53 and demonstrate resistance to melphalan^15^ were sensitive to sequential treatment in this study. Further, filanesib has been shown to be clinically effective in heavily pretreated patients with MM, including patients that had received treatment with novel agents. Since the effectiveness of conditioning regimens with combinations of novel agents and melphalan is dependent on drug sensitivity,^4^ sequential filanesib→melphalan conditioning may be more useful than lenalidomide/melphalan or bortezomib/melphalan particularly when HDT-ASCT is used as a salvage therapy in primary refractory or relapsed patients resistant to these agents. Our findings also suggest that sequential filanesib/melphalan could be a more effective ASCT conditioning regimen than the frequently employed HDT-melphalan regimen in which melphalan is administered two days prior to rescue (day 2) by ASCT (day 0). Although a potential limitation of any combination therapy is increased drug toxicity, the Fil→Mel combination may not be a problem since the main dose-limiting toxicities (DLT) for these two drugs do not overlap. As oral mucositis, the main DLT for melphalan is rarely observed for filanesib and cytopenia, the DLT for filanesib can be managed clinically via ASCT, the Fil→Mel combination would be expected to have an acceptable toxicity profile. Also, the synergistic interaction of the Fil→Mel regimen may allow for lower doses of melphalan to further reduce the incidence of oral mucositis. Given the pharmacokinetic profile of filanesib, with an extended plasma half-life (70–90 h),^7, 9^ a single dose of filanesib could be administered several days prior to HDT-melphalan, followed by subsequent ASCT. Indeed, two recent phase I/II clinical trials involving sequential treatment protocols for MM (lenalidomide for 7 days prior (day 8) to HDT melphalan (day 2),^4^ and busulfan for 4 days preceding HDT-melphalan on day-2 and bortezomib on days -2 and -1 followed by ASCT),^5^ demonstrated improved patient responses. Future studies will be important to determine the exact timing of filanesib treatment, as there is the possibility of delayed or failed engraftment given its myelosuppressive effects. Taken together, our study suggests that combination therapy with filanesib and melphalan warrants further study as a potential clinically relevant, intensive conditioning regimen for patients with multiple myeloma undergoing ASCT.

## Figures and Tables

**Figure 1:**
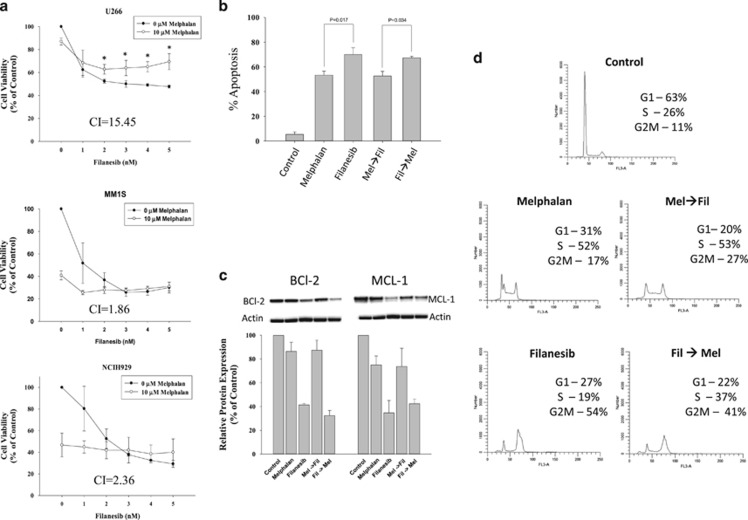
Cytotoxic effects of combination filanesib and melphalan is schedule dependent. (**a**) The human myeloma cell lines U266, MM1S and NCIH929 were treated with increasing doses of filanesib in the absence or presence of 10 μM melphalan for 72 h. At the end of treatment, cell proliferation was determined by MTS assay and reported as percent growth relative to control. The Combination Index (CI) calculated by the Chou-Talalay method was used to determine drug interaction. The CI reported is at doses of 10 μM melphalan and 5 nM filanesib. CI values >1.1 suggest antagonism. (**b**–**d**) U266 cells were treated with either melphalan (50 μM) for 1 h before 1.75 nM filanesib for an additional 48 h (Mel→Fil) or filanesib for 48 h with melphalan added to the treatment flasks after the first 24 h of filanesib treatment (Fil→Mel). (**b**) Apoptosis was determined by assessing the presence of apoptotic nuclei following staining with Hoechst/Propidium Iodide. (**c**) Whole-cell lysates were prepared at the end of treatment and immunoblots were performed using anti-MCL1 and anti-BCL2 antibodies. Relative intensities for BCL-2 and MCL-1 were normalized to β actin and plotted as percent of control. (**d**) Cell cycle distribution was determined by flow cytometry. Statistical analysis was performed with one-way ANOVA followed by Student Newman–Keuls *post hoc* test when significance was detected (*P*<0.05). Western blots and cell cycle distributions are representative of three individual experiments.

**Figure 2:**
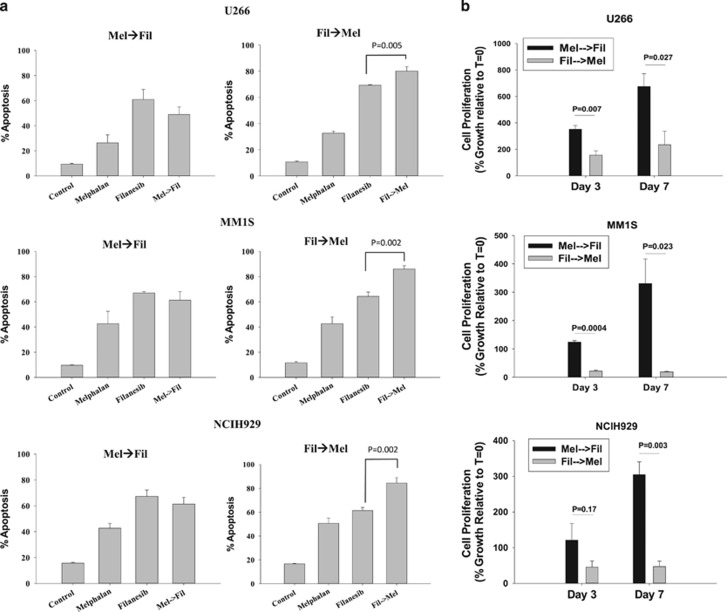
Fil→Mel sequential treatment significantly inhibits drug-free recovery of human myeloma cell lines. U266, MM1S and NCIH929 cells were treated with melphalan (10 μM) for 1 h followed by filanesib (1.75 nM) for 48 h (Mel→Fil) or inversely with filanesib for 48 h followed by 1 h melphalan (Fil→Mel). At the end of treatment, cells were resuspended in drug-free media and allowed to recover for 7 days. (**a**) Apoptosis was assessed 48 h after drug washout by Annexin V staining (*N*=3). Statistical analysis was performed by Two-way ANOVA for each treatment protocol independently. (**b**) Cellular proliferation was assayed on day 3 and day 7 following drug washout. For direct comparison of Mel→Fil and Fil→Mel treatment protocols, the percent growth from the beginning of the experiment was compared using a two-tailed Student's *t*-test (*N*=3). Statistical significance was set at *P*<0.05.

## References

[bib1] Usmani SZ, Crowley J, Hoering A, Mitchell A, Waheed S, Nair B et al. Improvement in long-term outcomes with successive Total Therapy trials for multiple myeloma: are patients now being cured? Leukemia 2013; 27: 226–232. 22705990 10.1038/leu.2012.160 PMC3744094

[bib2] Moreau P, Attal M, Facon T. Frontline therapy of multiple myeloma. Blood 2015; 125: 3076–3084. 25838345 10.1182/blood-2014-09-568915

[bib3] Bayraktar UD, Bashir Q, Qazilbash M, Champlin RE, Ciurea SO. Fifty years of melphalan use in hematopoietic stem cell transplantation. Biol Blood Marrow Transplant 2013; 19: 344–356. 22922522 10.1016/j.bbmt.2012.08.011 PMC4337224

[bib4] Shah N, Thall PF, Fox PS, Bashir Q, Shah JJ, Parmar S et al. Phase I/II trial of lenalidomide and high-dose melphalan with autologous stem cell transplantation for relapsed myeloma. Leukemia 2015; 29: 1945–1948. 25721897 10.1038/leu.2015.54 PMC4785590

[bib5] Rodriguez TE, Hari P, Stiff PJ, Smith SE, Sterrenberg D, Vesole DV. Busulfan, melphalan, and bortezomib versus high-dose melphalan as a conditioning regimen for autologous hematopoietic stem cell transplantation in multiple myeloma. Biol Blood Marrow Transplant 2016; 22: 1391–1396. 27164062 10.1016/j.bbmt.2016.03.021 PMC5075527

[bib6] Tunquist BJ, Woessner RD, Walker DH. Mcl-1 stability determines mitotic cell fate of human multiple myeloma tumor cells treated with the kinesin spindle protein inhibitor ARRY-520. Mol Cancer Ther 2010; 9: 2046–2056. 20571074 10.1158/1535-7163.MCT-10-0033

[bib7] Khoury HJ, Garcia-Manero G, Borthakur G, Kadia T, Foudray MC, Arellano M et al. A phase 1 dose-escalation study of ARRY-520, a kinesin spindle protein inhibitor, in patients with advanced myeloid leukemias. Cancer 2012; 118: 3556–3564. 22139909 10.1002/cncr.26664 PMC4984525

[bib8] Sagar Lonial M, Cohen A, Zonder J, Benzinger WI, Kaufman JL, Orlowski RZ et alIn The American Society of Hematology Annual Meeting and Exposition. New Orleans, LA, USA, 2013.

[bib9] LoRusso PM, Goncalves PH, Casetta L, Carter JA, Litwiler K, Roseberry D et al. First-in-human phase 1 study of filanesib (ARRY-520), a kinesin spindle protein inhibitor, in patients with advanced solid tumors. Invest New Drugs 2015; 33: 440–449. 25684345 10.1007/s10637-015-0211-0

[bib10] Chou TC, Martin N. Compusyn for Drug Combinations and for General Dose-Effect Analysis. ComboSyn: Paramus, NJ, USA, 2005.

[bib11] Vanhoefer U, Harstrick A, Wilke H, Schleucher N, Walles H, Schroder J et al. Schedule-dependent antagonism of paclitaxel and cisplatin in human gastric and ovarian carcinoma cell lines *in vitro*. Eur J Cancer 1995; 31A: 92–97. 7695986 10.1016/0959-8049(94)00440-g

[bib12] Doo NW, Thompson PA, Prince HM, Seymour JF, Ritchie D, Stokes K et al. Bortezomib with high dose melphalan conditioning for autologous transplant is safe and effective in patients with heavily pretreated and high risk multiple myeloma. Leuk Lymphoma 2013; 54: 1465–1472. 23121086 10.3109/10428194.2012.746682

[bib13] Paiva B, Vidriales MB, Montalban MA, Perez JJ, Gutierrez NC, Rosinol L et al. Multiparameter flow cytometry evaluation of plasma cell DNA content and proliferation in 595 transplant-eligible patients with myeloma included in the Spanish GEM2000 and GEM2005<65y trials. Am J Pathol 2012; 181: 1870–1878. 22974582 10.1016/j.ajpath.2012.07.020

[bib14] Chang H, Qi C, Yi QL, Reece D, Stewart AK. p53 gene deletion detected by fluorescence in situ hybridization is an adverse prognostic factor for patients with multiple myeloma following autologous stem cell transplantation. Blood 2005; 105: 358–360. 15339849 10.1182/blood-2004-04-1363

[bib15] Surget S, Lemieux-Blanchard E, Maiga S, Descamps G, Le Gouill S, Moreau P et al. Bendamustine and melphalan kill myeloma cells similarly through reactive oxygen species production and activation of the p53 pathway and do not overcome resistance to each other. Leuk Lymphoma 2014; 55: 2165–2173. 24308434 10.3109/10428194.2013.871277

